# Surface Plasmon Resonance Based Biosensors for Exploring the Influence of Alkaloids on Aggregation of Amyloid-β Peptide

**DOI:** 10.3390/s110404030

**Published:** 2011-04-06

**Authors:** Bartłomiej Emil Kraziński, Jerzy Radecki, Hanna Radecka

**Affiliations:** Institute of Animal Reproduction and Food Research of Polish Academy of Sciences, Tuwima 10 Street, 10-747 Olsztyn, Poland; E-Mails: b.krazinski@pan.olsztyn.pl (B.E.K.); j.radecki@pan.olsztyn.pl (J.R.)

**Keywords:** amyloid-β peptide, surface plasmon resonance, alkaloids, thioaliphatic acid monolayer

## Abstract

The main objective of the presented study was the development of a simple analytical tool for exploring the influence of naturally occurring compounds on the aggregation of amyloid-β peptide (Aβ_40_) in order to find potential anti-neurodegenerative drugs. The gold discs used for surface plasmon resonance (SPR) measurements were modified with thioaliphatic acid. The surface functionalized with carboxylic groups was used for covalent attaching of Aβ_40_ probe by creation of amide bonds in the presence of EDC/NHS. The modified SPR gold discs were used for exploring the Aβ_40_ aggregation process in the presence of selected alkaloids: arecoline hydrobromide, pseudopelletierine hydrochloride, trigonelline hydrochloride and α-lobeline hydrochloride. The obtained results were discussed with other parameters which govern the phenomenon studied such as lipophilicity/hydrophilicy and Aβ_40_-alkaloid association constants.

## 1. Introduction

Abnormal folding and aggregation of proteins characterizes many, if not all, neurodegenerative disorders such as Alzheimer’s (AD), Parkinson’s, Creutzfeldt-Jakob disease and large group of poliglutamine disorders including Huntington’s disease [1]. Aggregation of Amyloid-β peptide (Aβ) from its soluble monomers into insoluble well-ordered amyloid fibrils has been assumed to be a key molecular hallmark in the pathology of Alzheimer’s disease (AD) [2,3]. In a nucleation-dependent polymerization model Aβ_40_ aggregation consists of two phases: thermodynamically unfavourable nuclei formation, followed by their spontaneous elongation and growth. Soluble α-helical or random-coil structures can convert into β-sheet protofibrilar intermediates which act as a seed for amyloid formation and plaque precipitation [4,5]. Formation of soluble β-sheet oligomers has been reported to be essential for neurodegeneration processes and is suspected to play a principal role in the Aβ-mediated toxicity [6]. Thus targeting of α-helical and random-coil structures’ transition to β-sheeted species could provide AD prevention and treatment or retard the onset of disease. Among other modes of action, direct targeting Aβ misfolding and aggregation phenomenon might be one of the most promising therapeutic methods against AD disease.

Numerous plant-derived molecules have been tested *in vitro* and *in vivo* but special attention has been reserved for the alkaloid nicotine and its derivatives, as they are well known to exert an influence on cognitive functions [7]. While it seems that first of all the multiple influence of nicotine on cholinergic transmission is responsible for its beneficial effects on AD patients [8–10], there are *in vitro* studies suggesting that this alkaloid can alter Aβ amyloidogenesis directly [11–14]. The associations of aggregated Aβ_40_ with nicotine and cotinine as well as the other pyridine- and piperdine-derived alkaloids were determined electrochemically [15,16]. Since Aβ is a target molecule in many AD therapies, compounds revealing significant affinity towards this peptide could also serve as effective Aβ oligomerization and neurotoxicity inhibitors and have to be taken into consideration as possible anti-AD drugs.

Surface plasmon resonance (SPR) spectroscopy has been successfully employed in studies concerning molecular interactions associated with different amyloidoses [17]. Various aspects of Aβ oligomerization, fibril growth, dissociation as well as Aβ binding to biomolecules were examined with SPR [18]. The *in vitro* anti-amyloidogenic activity of metal ions chelators, short peptides, antibody fragments and flavonoids has been tested utilizing SPR-based methods [19–23]. In most SPR-based biosensors applied for study of Aβ aggregation as well as its interactions with aggregation inhibitors, carboxymethyldextran was used as the interaction platform [20–24].

In the present study we proposed a simple in preparation SPR biosensor based on a thioaliphatic acid self-assembled monolayer (SAM) formed on gold SPR disc. The attachment of Aβ_40_ peptide probe to the carboxyl-terminated surface was performed via amide bond formation.

**Figure 1.**
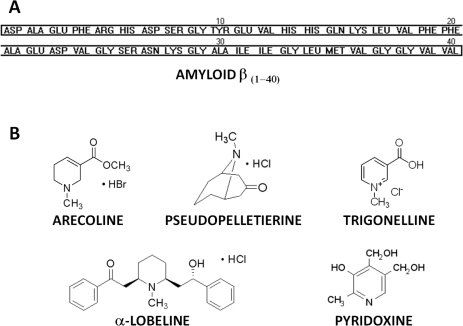
Amyloid β peptide (1–40) amino acid sequence (**A**) and chemical structure of tested compounds (**B**).

The proposed biosensor was applied for *in situ* monitoring of Aβ_40_ early aggregation stages (oligomerization) in the presence of potential aggregation inhibitors. The influence of arecoline, α-lobeline, trigonelline and pseudopelletierine (Figure 1(B)) on Aβ_40_ aggregation was examined. These compounds derived from domestic plants are either commonly used as psychoactive substances (arecoline and α-lobeline) or are consumed as a dietary component (trigonelline and pseudopelletierine). Plants and products containing the considered alkaloids are easily available, which creates the possibility of using them (or their derivatives) as a diet supplementation in AD prevention and treatment. For comparison, one pharmacologically unrelated compound, pyridoxine (vitamin B_6_) was tested too.

## 2. Experimental Section

### 2.1. Chemicals

Amyloid β protein (1–40), trifluoroacetate salt Aβ_40_ was purchased from Bachem, Switzerland (lot 1020370). Ammonium hydroxide, 11-mercaptoundecanoic acid (11-MUA), trifluoroacetic acid, 2-morpholinoethanesulfonic acid (MES), *N*-hydroxysuccinimide (NHS), *N*-(3-dimethylaminopropyl)-*N*’-ethylcarbodiimide hydrochloride (EDC), ethanolamine, pyridoxine, arecoline hydrobromide, pseudopelletierine hydrochloride, trigonelline hydrochloride, α-lobeline hydrochloride and PBS components: sodium chloride, potassium chloride, sodium phosphate dibasic, potassium phosphate monobasic were supplied by Sigma-Aldrich, Poznań, Poland. Ethanol absolute, hydrochloric acid and sodium hydroxide were obtained from POCH, Poland. Reagents and solvents were of analytical grade and used without any purification step. All aqueous solutions were prepared using autoclaved Milli-Q water, resistivity 18.2 MΩ·cm (Millipore Corporation, USA). All buffers used for SPR experiments were rigorously filtrated through 0.22 μm sterile PVDF filters (Roth, Germany).

### 2.2. Surface Plasmon Resonance Apparatus

An Autolab Springle SPR system (Eco Chemie, The Netherlands) equipped with a thermostatic water bath (Julabo Labortechnik, Germany) was used. Springle SPR was employed for monitoring both Aβ_40_ probe immobilization and Aβ_40_ aggregation. The experiments were carried out continuously and the immobilization step was followed by aggregation procedure, as described below.

### 2.3. Peptide Preparation

The lyophilized synthetic Aβ_40_ peptides were dissolved in 0.02% ammonia solution at the concentration of 300 μM by brief vortexing. Then the solution was sonicated for 2 min in a water bath at 4 °C. The Aβ_40_ aliquots were stored in at −85 °C.

### 2.4. Formation of 11-MUA SAM

Gold-coated SPR discs were supplied by Eco Chemie, The Netherlands. Prior to modification, discs were rinsed with Milli-Q water then with ethanol and dried thoroughly in a stream of nitrogen. Gold discs were treated with UV-ozone chamber (Novascan, USA) for 20 min in order to remove any organic contaminations. Clean gold SPR discs were immediately immersed for 30 min in 1.0 mM solution of 11-MUA in ethanol containing 2% of trifluoroacetic acid. Afterwards discs were rinsed with ethanol, 10% solution of ammonia in ethanol, again with ethanol and gently dried in a stream of nitrogen before placing in SPR chamber. Formation of 11-MUA SAM was carried out according to modified method described by Wang *et al*. [25].

### 2.5. Immobilization Aβ_40_ Peptide Probe

Probe immobilization was performed at 22 °C using 50 mM MES (pH 5.5) as running buffer. Freshly 11-MUA-modified discs were placed in the SPR chamber and conditioned in running buffer for 2 h. Every 30 min discs were washed with 4 mL of running buffer. After conditioning, when stable readout was achieved, the carboxyl groups of 11-MUA SAM were activated by treating with a mixture of 100 mM EDC and 50 mM NHS in 50 mM MES (pH 5.5) for 15 min. Subsequently discs were washed with 2 mL of running buffer and the value of SPR angle shift was recorded after 3 min. Aβ_40_ aliquots were diluted with 50 mM MES (pH 6.5) to 30μM, briefly vortexed and injected on the activated disc surface. After 20-min incubation peptide solution was rinsed off with 2 mL of running buffer and followed by quenching the remaining NHS-activated sites with 1.0 M ethanolamine in 50 mM MES (pH 8.5) for 10 min. Finally, discs were rinsed with 4 mL of the running buffer and the value of SPR angle shift was recorded 10 min after the end wash.

### 2.6. Aβ_40_ Aggregations

Aggregation experiments were performed at 37 °C using 10 mM PBS (pH 7.4) as running buffer. Prior to the experiment, discs with immobilized probe were washed with 4 mL of running buffer and conditioned until the stable readout was gained. SPR angle shift values were recorded after 1 h of conditioning. Aβ_40_ aliquots were diluted within PBS to 30 μM. 1.0 mM alkaloids: arecoline, pseudopelletierine, trigonelline and α-lobeline as well as pyridoxine were dissolved in PBS. Final pH was adjusted to 7.4 ± 0.05. Then discs were incubated in a solution of Aβ_40_ with a supplementation of selected alkaloid for 2 h followed by washing with 4 mL of running buffer. The same experiment, but without tested compounds has been preformed too (control aggregation). Values of SPR shift angle were recorded 20 min after the end wash.

### 2.7. Aggregation of Aβ_40_ Peptide on the Surface of 11-MUA SAM Treated with Ethanolamine after Activation with EDC/NHS (Background Aggregation)

Freshly 11-MUA-modified discs were placed in SPR chamber and conditioned in 50 mM MES (pH 5.5) running buffer for 2 h. Every 30 min discs were washed with 4 mL of running buffer. After conditioning, when stable readout was achieved, the carboxyl groups of 11-MUA SAM were activated by treating with a mixture of 100 mM EDC and 50 mM NHS in 50 mM MES (pH 5.5) for 15 min. Subsequently discs were washed with 2 mL of running buffer and the value of SPR angle shift was recorded after 3 min. Instead of diluted Aβ_40_ peptide, a solution containing 50 mM MES (pH 5.5) with 0.002% ammonia only was injected, incubated for 20-min and rinsed off with 2 mL of running buffer. This was followed by quenching the remaining NHS-activated sites with 1.0 M ethanolamine in 50 mM MES (pH 8.5) for 10 min. Finally, discs were rinsed with 4 ml of the running buffer and the value of SPR angle shift was recorded 10 min after the end wash. Aggregation was performed at 37 °C using 10 mM PBS (pH 7.4) as running buffer. Prior to the experiment, discs were washed with 4 mL of running buffer and conditioned until the stable readout was gained. SPR angle shift values were recorded after 1 h of conditioning. Then discs were incubated in a solution of 30 μM Aβ_40_ in PBS for 2 h followed by washing with 4 mL of running buffer. Values of SPR shift angle were recorded 20 min after the end wash.

### 2.8. Influence of Tested Alkaloids on SPR Angle Shift of Aβ_40_-Funcionalized Disc

Experiments were performed at 37 °C using 10 mM PBS (pH 7.4) as running buffer. Prior to the experiment, discs with immobilized probe were washed with 4 mL of running buffer and conditioned until the stable readout was gained. SPR angle shift values were recorded after 1 h of conditioning. 1.0 mM alkaloids: arecoline, pseudopelletierine, trigonelline and α-lobeline as well as pyridoxine were dissolved in PBS containing 0.002% ammonia solution. Final pH was adjusted to 7.4 ± 0.05. Then discs were incubated in alkaloid solution for 2 h followed by washing with 4 mL of running buffer. Values of SPR shift angle were recorded 20 min after the end wash.

### 2.9. Data Presentation and Statistical Analysis

Aggregation assay results were presented as means ± standard deviation for at least five replicates for each experimental group. Results were submitted to the t-test (The Analysis ToolPak, Microsoft, USA) and values of p < 0.05 were considered as statistically significant.

## 3. Results and Discussion

**Figure 2.**
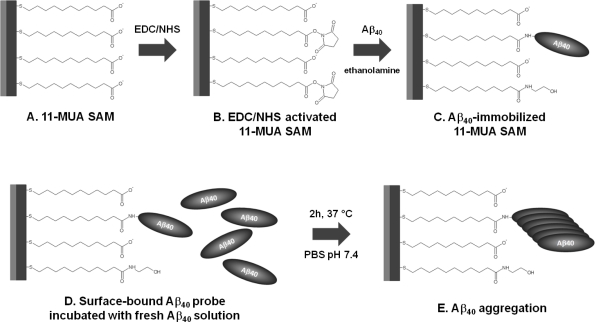
Scheme of Aβ_40_ probe immobilization on 11-MUA-modified surface of SPR gold disc (**A–C**) and Aβ_40_ aggregation assay on Aβ_40_-functionalized gold discs surface (**D–E**).

**Figure 3.**
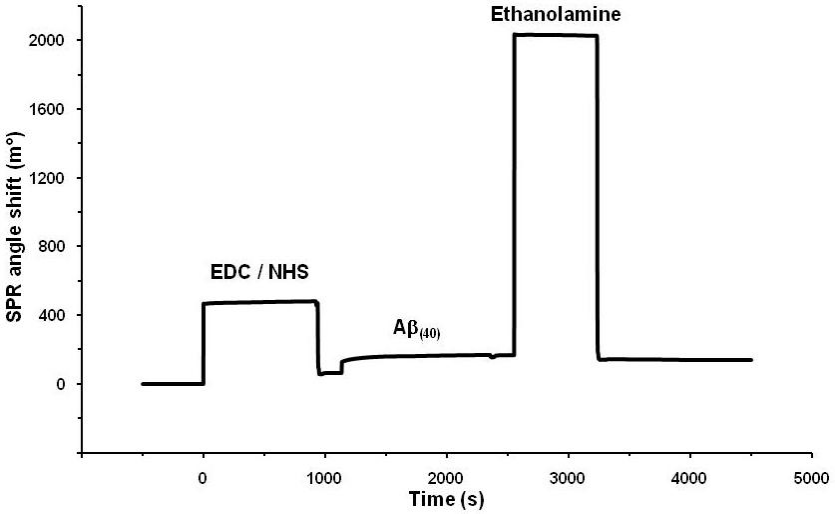
SPR binding curve for Aβ_40_ immobilization on 11-MUA-SAM modified gold disc (For measurement details, see Experimental part 2.5).

The 11-MUA-modified SPR gold discs were utilized for the covalent binding of Aβ_40_ peptide probe. The immobilization procedure scheme is shown in Figure 2. The immobilization of Aβ_40_ peptide probe, performed via EDC/NHS coupling chemistry [26], was monitored constantly and the SPR angle shift was recorded. The representative binding curve was illustrated in Figure 3. The probe-immobilizing procedure resulted in around 0.55 ± 0.12 ng of Aβ_40_ peptide attached per 1 mm^2^ of disc surface.

**Figure 4.**
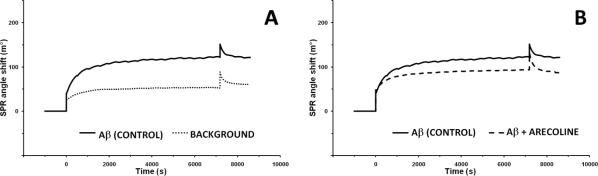

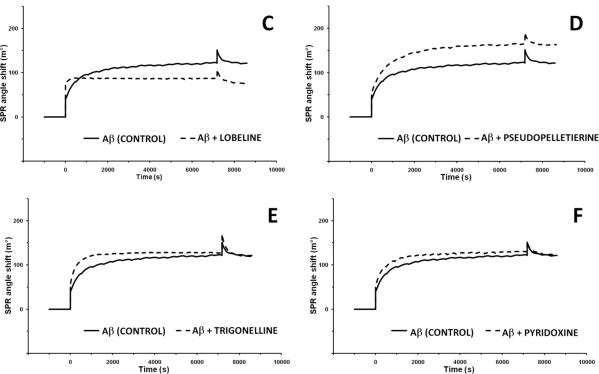
The representative SPR binding curves for Aβ_40_ aggregations. (**A**) Aggregations of Aβ_40_ injected alone performed on SPR gold discs surface functionalized with: Aβ_40_ probe (control) or with 11-MUA SAM treated with ethanolamine after activation with EDC/NHS (background); (**B–F**) Aggregations of Aβ_40_ injected alone (control) or in the presence of 1.0 mM of tested compound performed on a Aβ_40_-functionalized gold discs surface (For measurement conditions-see Experimental parts: 2.6 and 2.7).

In order to check if the proposed biosensor is able to detect the early steps of the aggregation process, two series of control aggregations were carried out. In first, the Aβ_40_ solution was injected on the surface of Aβ_40_ functionalized disc (control aggregation), and in second, the same solution was injected on the disk surface modified only with 11-MUA SAM, which was treated with ethanolamine after activation with EDC/NHS (background aggregation). The value of SPR angle shifts for control aggregation was 125.7 ± 15.6 m° and for background only 60.1 ± 3.6 m° (Figure 4(A)). This indicated that the rate of specific deposition of Aβ_40_ on surface modified with Aβ_40_-probe was *ca*. 50% higher than non-specific background deposition of Aβ_40_ on the ethanolamine deactivated 11-MUA SAM. The phenomenon of beta-amyloid aggregation on the surface of SAMs with different functional groups (without Aβ_40_-probe) was reported in the literature [27]. The authors concluded that SAMs induced aggregation of Aβ. The hybrophobicity/hydrophilicy of functional groups present on the SAMs’ surface is the main factor which governs this phenomenon. Molecular docking and dynamics simulations have been applied for exploring the conformational changes and adsorption behaviour of Aβ monomer on various SAMs. The hydrophobic CH_3_-SAM has a lower energy barrier for Aβ monomer adsorption than OH-SAM [28]. For the COOH-SAM and NH_2_-SAM bearing relatively large charge in the head groups, electrostatic interactions between Aβ and SAMs provide the additional driving force for Aβ adsorption [29]. The background Aβ aggregation was performed on the 11-MUA SAM, treated with ethanolamine after activation with EDC/NHS. Thus, on this surface the OH groups are dominant. Only a few COOH could be present, so Aβ monomer adsorption on such SAM is driven by very weak Aβ-SAM interactions with strong SAM-water interactions which results in a net weak affinity of Aβ adsorption on OH-SAM [29]. Prior to aggregation assays with alkaloids and pyridoxine, possible interactions between those compounds and the Aβ_40_-functionalized SPR gold disc were checked. Incubations of probe-modified discs with the tested alkaloids injected alone onto disc surface revealed that SPR angle shifts were negligible (−0.8 m° for arecoline, −0.6 m° for α-lobeline, 0.5 m° for pseudopelletierine, −0.3 m° for trigonelline and 0.5 m° for pyridoxine). Thus, the proposed assay was deemed selective and suitable for performing the Aβ_40_ aggregation processes in the presence of selected compounds.

Figure 4(B–E) shows the representative binding curves for incubation of Aβ_40_-probe-functionalized gold discs with Aβ_40_ solution containing 1.0 mM of tested alkaloid. For comparison the representative binding curve for incubation of Aβ_40_-probe-functionalized disc with Aβ_40_ solution without any additives (control aggregation) was included.

The addition of α-lobeline and arecoline to the assay significantly (p < 0.01) decreased Aβ_40_ aggregation. Values of SPR angle shifts decreased from 125.7 ± 15.6 m° for control incubation (Aβ_40_ injected alone) to 83.1 ± 16.5 m° for arecoline (Figure 4(B)) and 74.8 ± 23.8 m° for α-lobeline supplementation (Figure 4(C)). On the contrary, addition of pseudopelletierine to the aggregation assay showed a tendency to elevate SPR angle shift to 164.5 ± 14.1 m° (Figure 4(D)). Trigonelline did not affect Aβ_40_ peptide deposition and SPR angle shift resulted in 121.9 ± 25.6 m° (Figure 4(E)). Aggregation assay with addition of pharmaceutically unrelated compound, pyridoxine (vitamin B_6_), did not influence Aβ_40_ deposition (122.5 ± 14.4 m°; Figure 4(F)).

**Figure 5.**
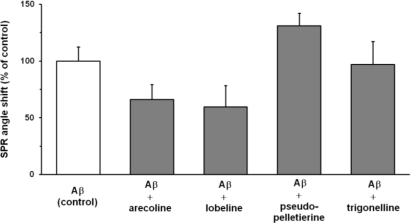
SPR angle shift values for Aβ_40_ aggregation on an Aβ_40_-functionalized gold discs. Measurement conditions: see Experimental part 2.6; values are significantly different (P < 0.01); N = 5.

The values of SPR angle shift was recorded 20 min after the final wash. Aβ_40_ deposition obtained for incubations with tested compounds was calculated relative to the aggregation control (100%) (Figure 5).

In our previous study, association constants for Aβ_40_ peptide and selected alkaloids were established electrochemically [16]. The highest affinity to the Aβ_40_ was determined for arecoline: 2.30 × 10^8^ M^−1^. The association constant for α-lobeline was equal to 0.90 × 10^8^ M^−1^, while binding of pseudopelletierine (0.57 × 10^8^ M^−1^) and trigonelline (0.28 × 10^8^ M^−1^) were relatively weaker [16]. In the present study, only two alkaloids: α-lobeline and arecoline, were shown to be potential inhibitors of early Aβ_40_ aggregation and their therapeutic and/or prophylactic significance can be suggested.

The phenomenon of interaction between examined compounds and Aβ peptide, resulting in altered aggregation, can be considered as a process composed of two steps. At the first stage alkaloid passes through the water-peptide interface, moving from aqueous phase to the relatively lipophilic environment consisted of Aβ molecules, either deposited on gold disc surface or remaining in the solution. In that case the permeability of substance is determined by its lipophilicity. This attribute also governs drug bioavailability and cannot be neglected when potential therapeutic or prophylactic benefits of compound administration are considered. Thus, the lipophilicity of drug candidate is an important factor which enables any subsequent ligand-Aβ_40_ peptide interactions. The second step of considered phenomenon refers to the direct interaction between the drug-and receptor site in the peptide. These two factors, ability to cross the interface and binding to the specific fragment of the peptide can be crucial for determining the effects of alkaloid influence on Aβ_40_ aggregation observed herein.

In light of those assumptions, the lipophilicity of tested compounds was taken into account. Log D (pH 7.4) values of the examined alkaloids and pyridoxine were found in ChemSpider online database [30] and was collected in Table 1. Log D is an octanol/water partition coefficient, where the neutral species as well as ionized forms are considered. It is assumed to be accurate for the prediction of molecule hydrophilic or lipophilic behaviour under given pH [31]. The theoretical log D values revealed that arecoline and α-lobeline are the most lipophilic among the studied alkaloids. Their log D values are equal to 0.71 and 2.14 respectively. Both of them appear to be inhibitors of Aβ_40_ aggregation.

The log D values of pseudopelletierine and trigonelline are −0.96 and −3.31, respectively, showing the predominantly hydrophilic character of these compounds under physiological pH. Pseudopelletierine demonstrated similar affinity towards Aβ_40_ peptide as was recorded for trigonelline or α-lobeline [16], but at the same time, this compound induced an acceleration of the Aβ_40_ aggregation process. The specific non-flat geometry of pseudopelletierine molecule (Figure 1) could be responsible for the observed effect. The results we have got for trigonelline indicated that the hydrophilicity of this compounds is a decisive parameter governing its very weak interaction with Aβ_40_ peptide. Similar results were observed for pyridoxine (vitamin B_6_; log D = −1.45) which is known as a neutral compound concerning Aβ_40_ peptide aggregation.

Protein aggregation processes have been widely explored by fluorescence assays [14,21,23,32], circular dichroism spectroscopy [11,22], atomic force microscopy [21,23,27,33] electrochemical methods [15,16,34] as well as SPR based biosensors [19–24,26,35–37]. The development of SPR techniques improved crucially the performance of bio-chemical sensing devices [38–40].

Here, we present the simple thioaliphatic carboxylic acid monolayer deposited in the one step of modification on the surface of SPR gold disc. Such a simple modification was suitable for covalent attaching of Aβ_40_-peptide probe via amide bond formation. It is worth underlining that the surface of 11-MUA SAM displayed good selectivity. The unspecific aggregation of Aβ_40_ was significantly lower in the comparison to the Aβ_40_ aggregation performed on the surface with Aβ_40_ probe covalently bound via amide bonds (Figure 4(A)).

## 4. Conclusions

The proposed SPR biosensor based on 11-mercaptoundecanoic acid (11-MUA) was useful for study of the influence of several alkaloids—arecoline hydrobromide, pseudopelletierine hydrochloride, trigonelline hydrochloride, α-lobeline hydrochloride—on the aggregation process of Aβ_40_-peptide. The measurement system was selective. Pyridoxine (vitamin B_6_), which is known as a neutral compound for Aβ_40_-peptide aggregation, displayed no influence on this phenomenon when studied by the analytical tool proposed. The obtained results showed that developed SPR-based biosensors could be applied for monitoring of Aβ_40_ aggregation phenomena in real-time, without using any chemical markers influencing the process.

## Figures and Tables

**Table 1.** Aβ_40_ aggregation in relation to control (100%), log *D* (pH 7.4) values of alkaloids [30] and their association constants with Aβ_40_ peptide [16].

**Compound**	**Aggregation [%]of control**	**Log D pH 7.4 [30]**	**Log K_a_ 10^8^ [M^−1^] [16]**
Lobeline	59.1 ± 18.9	2.14	0.90
Arecoline	66.1 ± 13.1	0.71	2.30
Pseudopelletierine	130.9 ± 11.2	−0.96	0.57
Trigonelline	97.0 ± 20.4	−3.31	0.28
Pyridoxine	97.5 ± 11.4	−1.45	-
